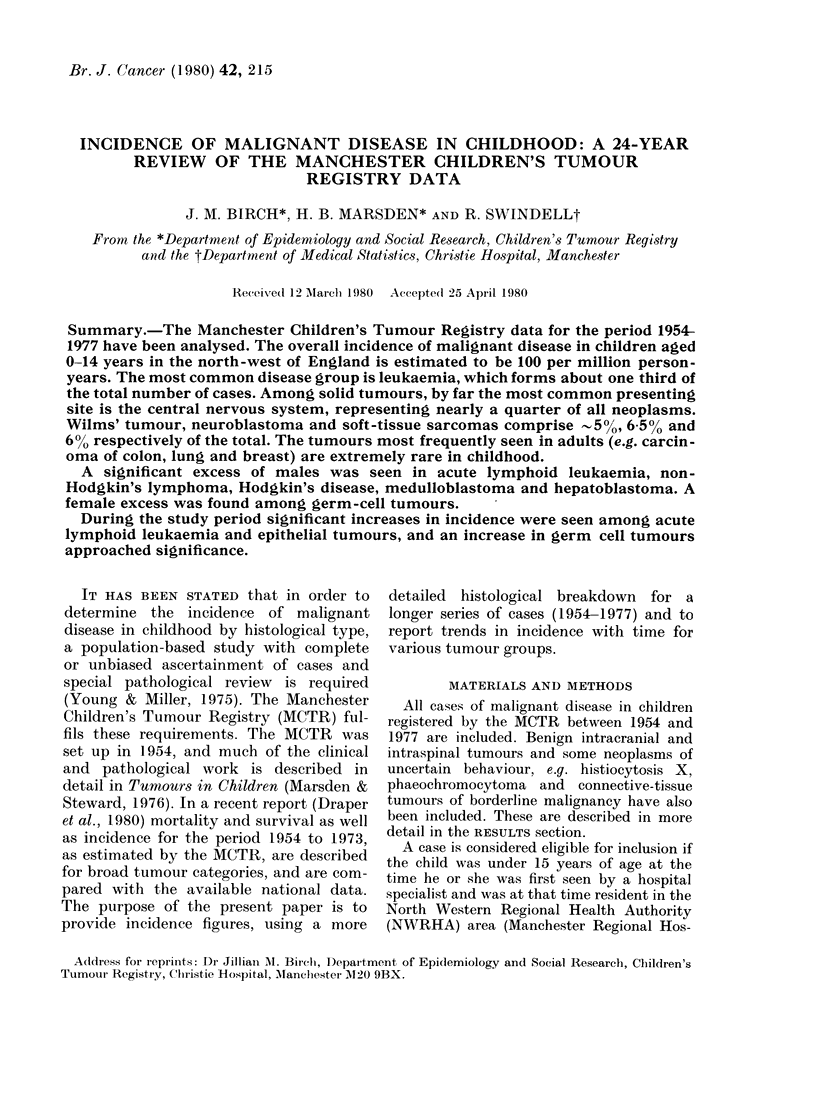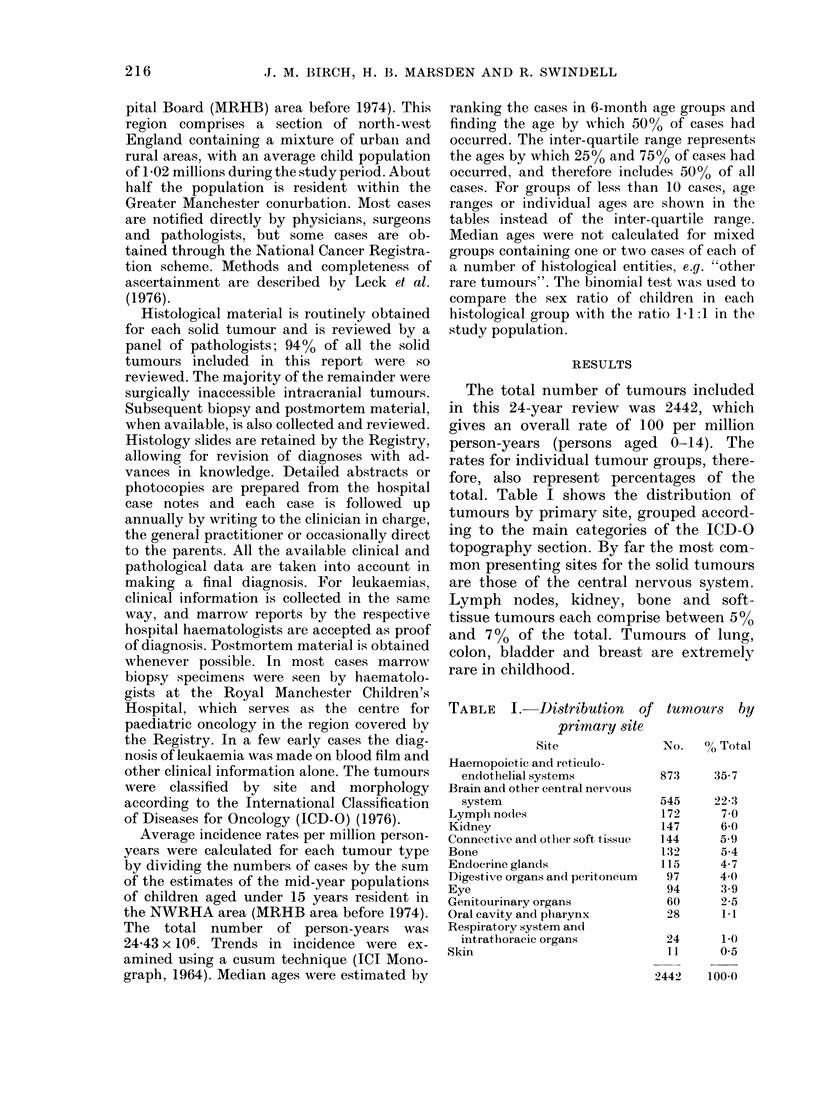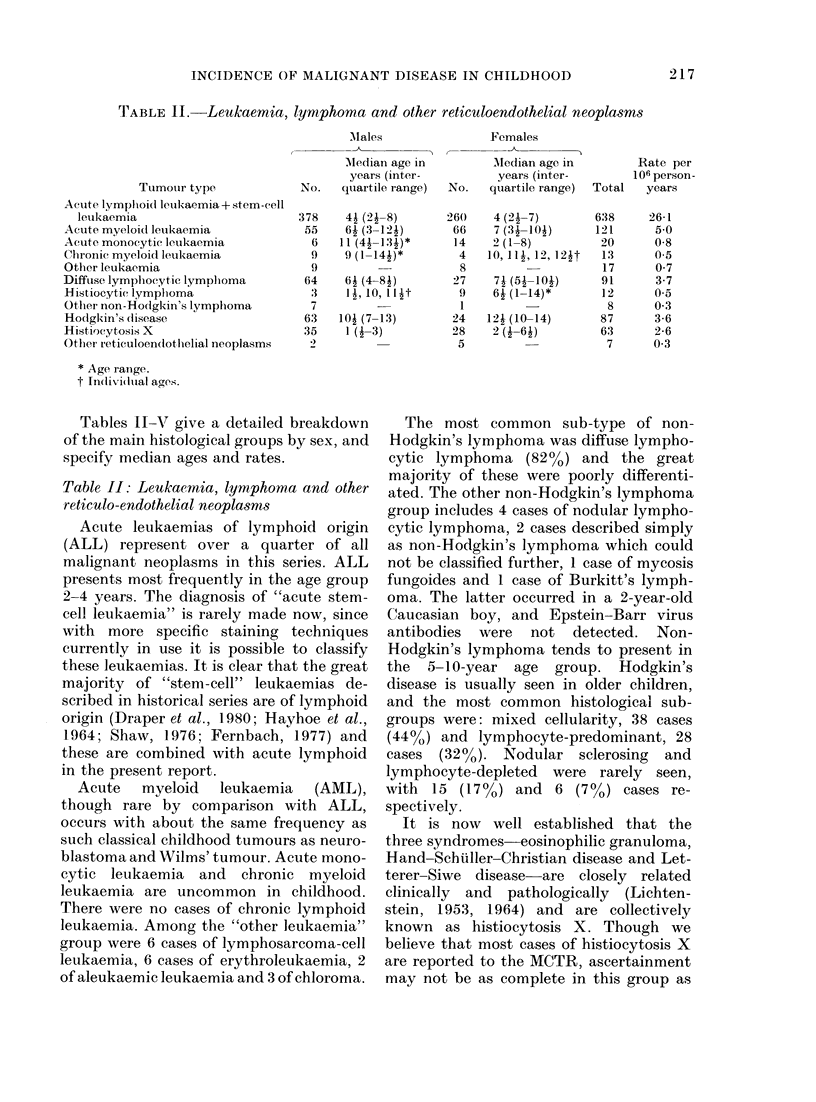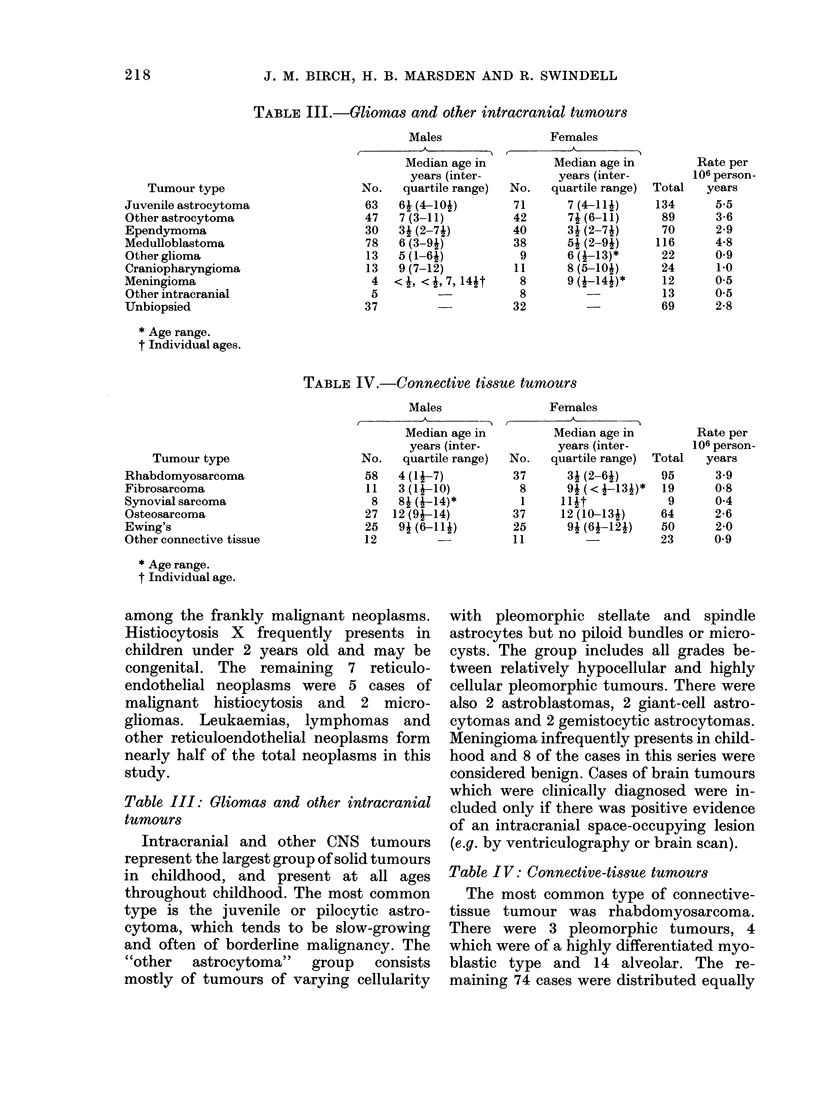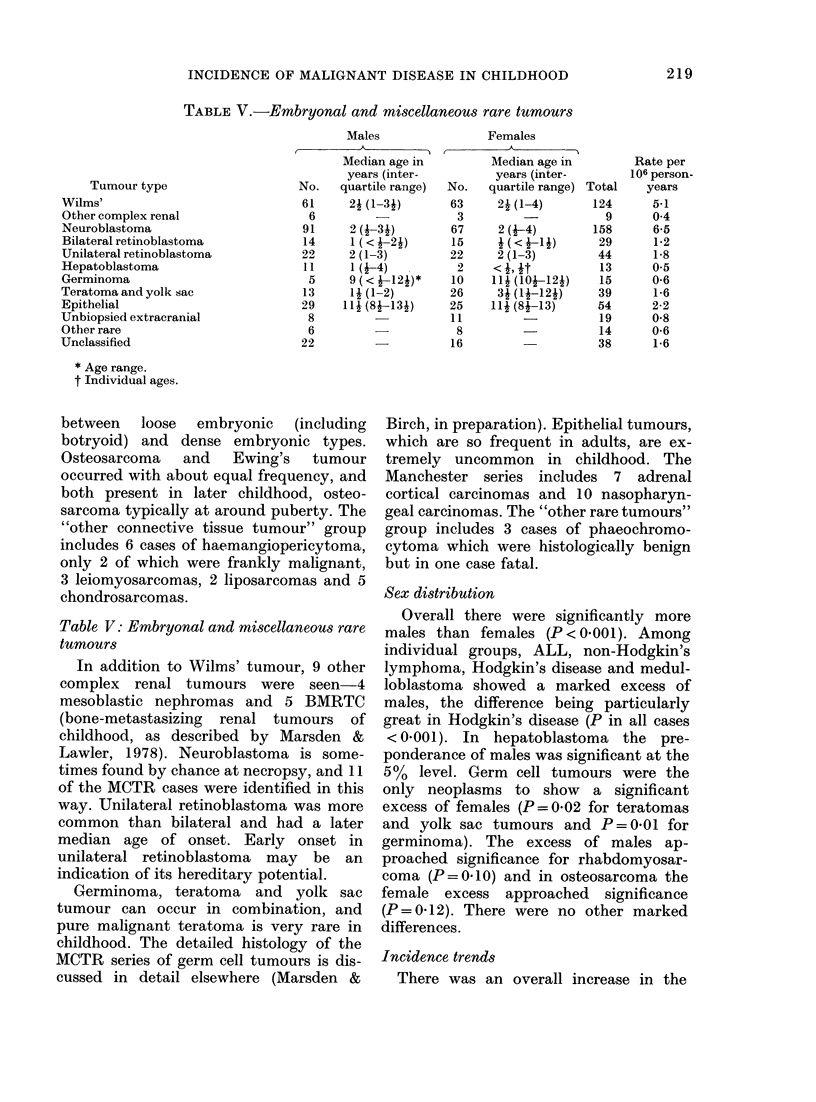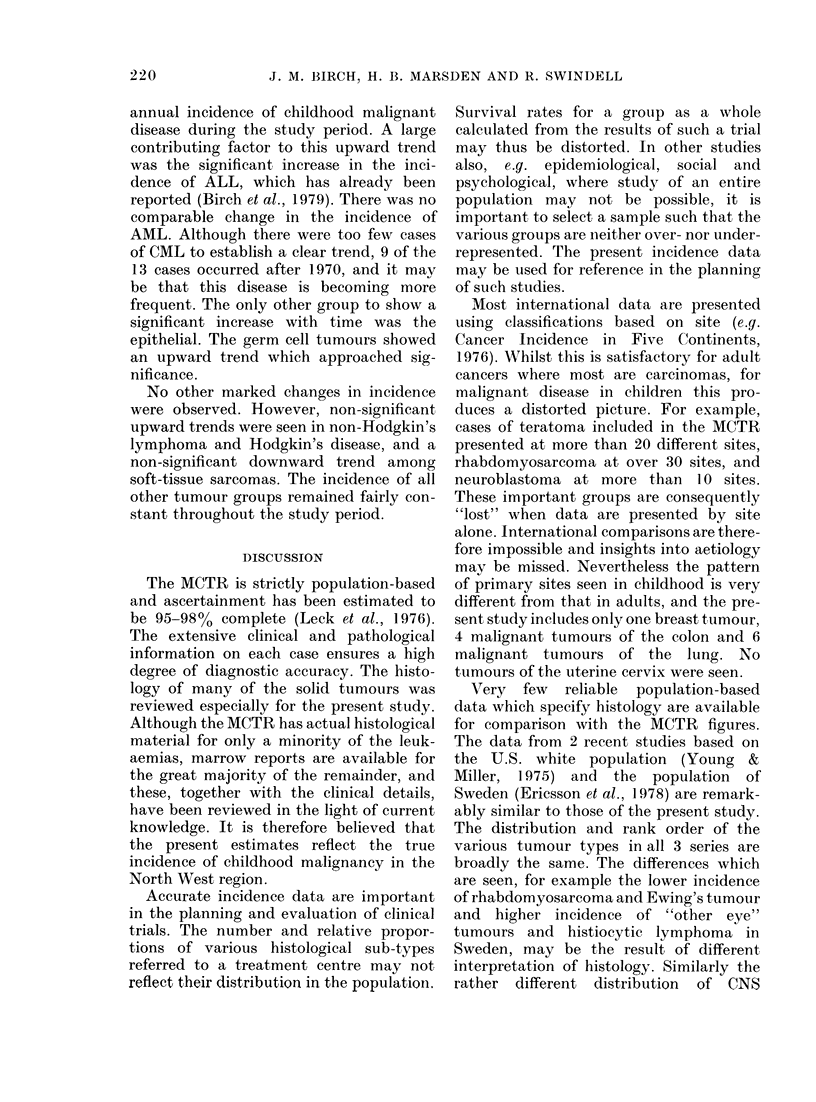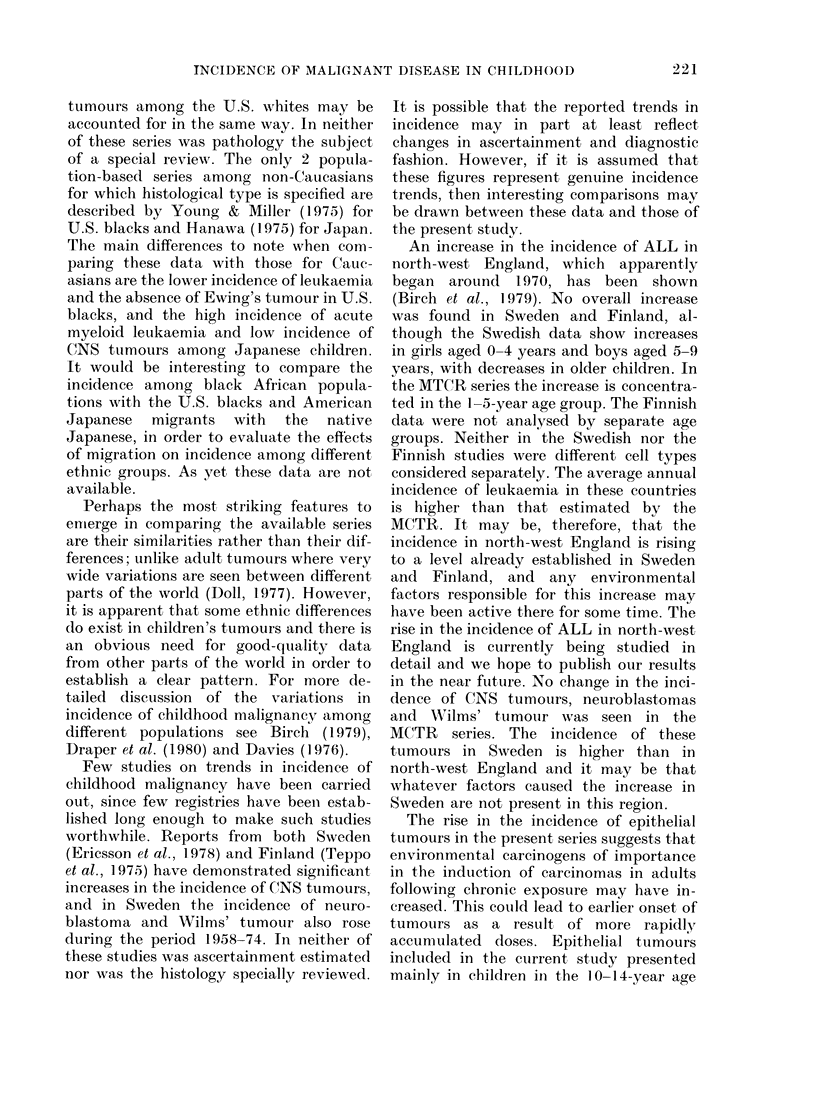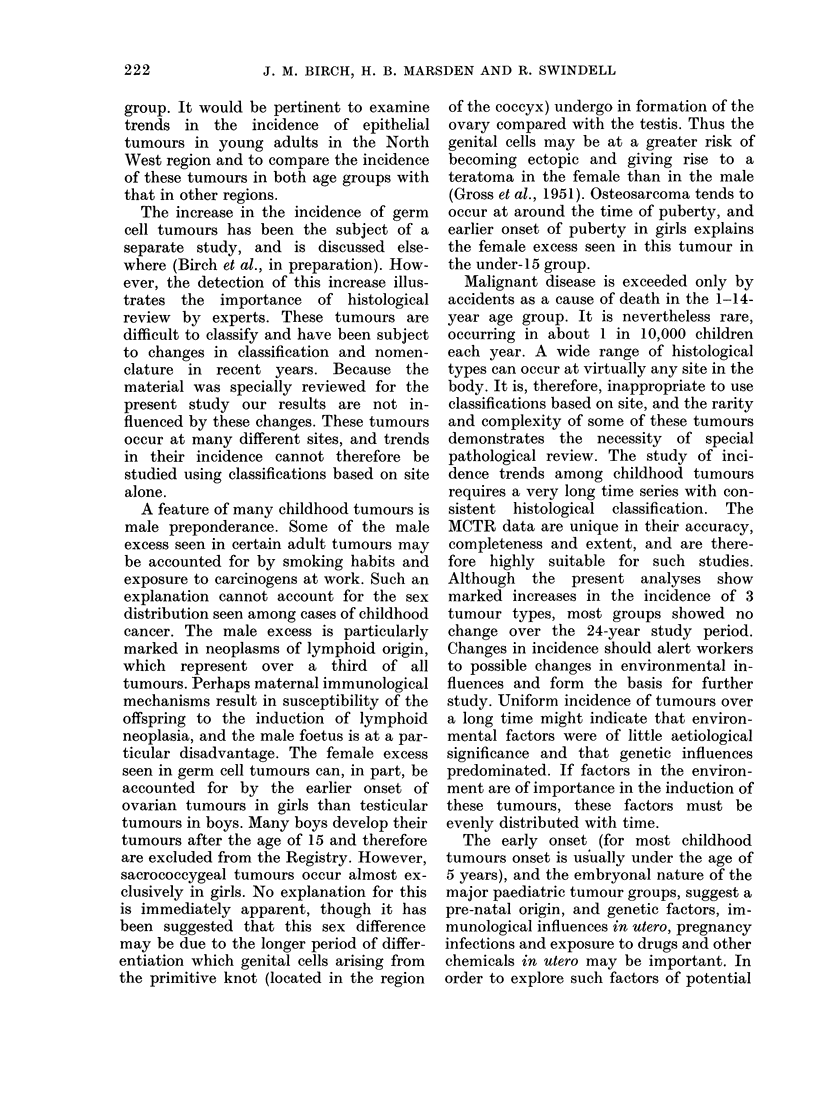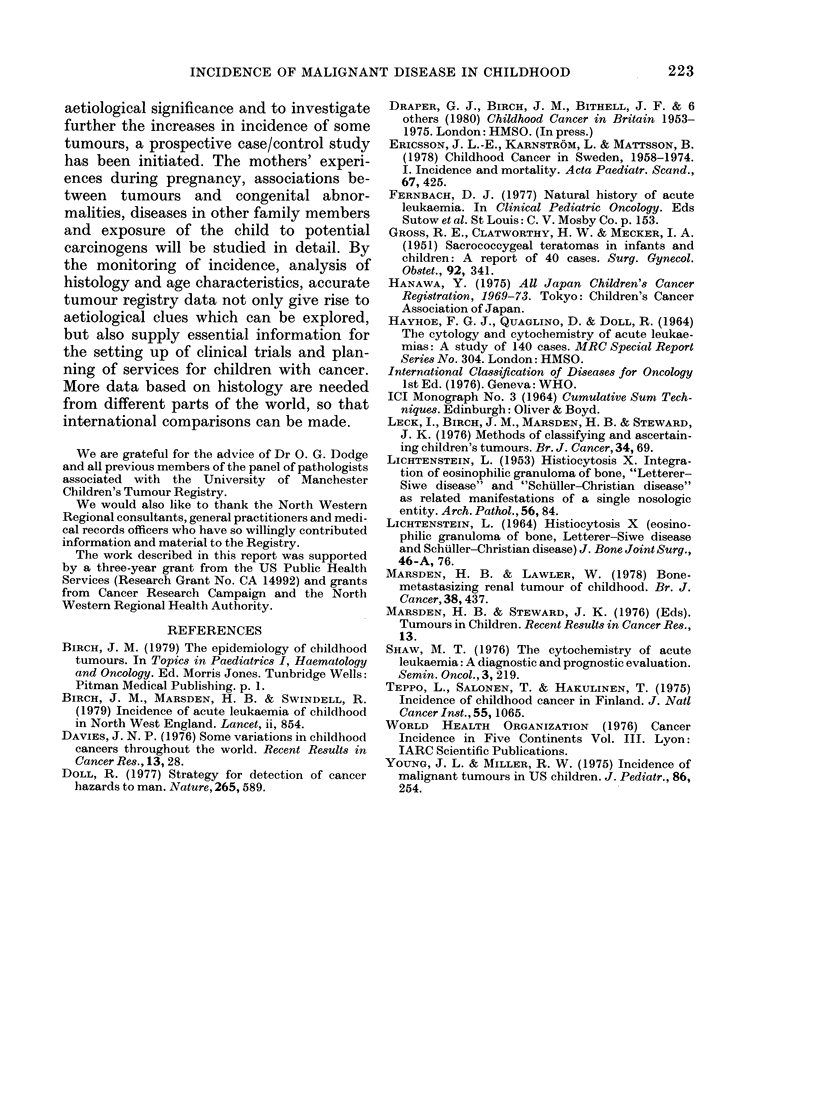# Incidence of malignant disease in childhood: a 24-year review of the Manchester Children's Tumour Registry data.

**DOI:** 10.1038/bjc.1980.221

**Published:** 1980-08

**Authors:** J. M. Birch, H. B. Marsden, R. Swindell

## Abstract

The Manchester Children's Tumour Registry data for the period 1954-1977 have been analysed. The overall incidence of malignant disease in children aged 0-14 years in the north-west of England is estimated to be 100 per million person-years. The most common disease group is leukaemia, which forms about one third of the total number of cases. Among solid tumours, by far the most common presenting site is the central nervous system, representing nearly a quarter of all neoplasms. Wilms' tumour, neuroblastoma and soft-tissue sarcomas comprise approximately 5%, 6.5% and 6% respectively of the total. The tumours most frequently seen in adults (e.g. carcinoma of colon, lung and breast) are extremely rare in childhood. A significant excess of males was seen in acute lymphoid leukaemia, non-Hodgkin's lymphoma, Hodgkin's disease, medulloblastoma and hepatoblastoma. A female excess was found among germ-cell tumours. During the study period significant increases in incidence were seen among acute lymphoid leukaemia and epithelial tumours, and an increase in germ cell tumours approached significance.


*Br. J. Cancer* (1980) **42,** 215

### INCIDENCE OF MALIGNANT DISEASE IN CHILDHOOD: A 24-YEAR REVIEW OF THE MANCHESTER CHILDREN'S TUMOUR REGISTRY DATA

J. M. BIRCH*, H. B. MARSDEN* AND R. SWINDELL†

*From the *Department of Epidemiology and Social Research, Children's Tumour Registry and the †Department of Medical Statistics, Christie Hospital, Manchester*

Received 12 March 1980 Accepted 25 April 1980

**Summary.—The Manchester Children's Tumour Registry data for the period 1954–1977 have been analysed. The overall incidence of malignant disease in children aged 0–14 years in the north-west of England is estimated to be 100 per million person-years. The most common disease group is leukaemia, which forms about one third of the total number of cases. Among solid tumours, by far the most common presenting site is the central nervous system, representing nearly a quarter of all neoplasms. Wilms' tumour, neuroblastoma and soft-tissue sarcomas comprise ~5%, 6·5% and 6% respectively of the total. The tumours most frequently seen in adults (*e.g.* carcinoma of colon, lung and breast) are extremely rare in childhood.**

**A significant excess of males was seen in acute lymphoid leukaemia, non-Hodgkin's lymphoma, Hodgkin's disease, medulloblastoma and hepatoblastoma. A female excess was found among germ-cell tumours.**

**During the study period significant increases in incidence were seen among acute lymphoid leukaemia and epithelial tumours, and an increase in germ cell tumours approached significance.**

IT HAS BEEN STATED that in order to determine the incidence of malignant disease in childhood by histological type, a population-based study with complete or unbiased ascertainment of cases and special pathological review is required (Young & Miller, 1975). The Manchester Children's Tumour Registry (MCTR) fulfils these requirements. The MCTR was set up in 1954, and much of the clinical and pathological work is described in detail in *Tumours in Children* (Marsden & Steward, 1976). In a recent report (Draper *et al.*, 1980) mortality and survival as well as incidence for the period 1954 to 1973, as estimated by the MCTR, are described for broad tumour categories, and are compared with the available national data. The purpose of the present paper is to provide incidence figures, using a more detailed histological breakdown for a longer series of cases (1954–1977) and to report trends in incidence with time for various tumour groups.

### MATERIALS AND METHODS

All cases of malignant disease in children registered by the MCTR between 1954 and 1977 are included. Benign intracranial and intraspinal tumours and some neoplasms of uncertain behaviour, *e.g.* histiocytosis X, phaeochromocytoma and connective-tissue tumours of borderline malignancy have also been included. These are described in more detail in the RESULTS section.

A case is considered eligible for inclusion if the child was under 15 years of age at the time he or she was first seen by a hospital specialist and was at that time resident in the North Western Regional Health Authority (NWRHA) area (Manchester Regional Hos-

Address for reprints: Dr Jillian M. Birch, Department of Epidemiology and Social Research, Children's Tumour Registry, Christie Hospital, Manchester M20 9BX.

pital Board (MRHB) area before 1974). This region comprises a section of north-west England containing a mixture of urban and rural areas, with an average child population of 1·02 millions during the study period. About half the population is resident within the Greater Manchester conurbation. Most cases are notified directly by physicians, surgeons and pathologists, but some cases are obtained through the National Cancer Registration scheme. Methods and completeness of ascertainment are described by Leck *et al.* (1976).

Histological material is routinely obtained for each solid tumour and is reviewed by a panel of pathologists; 94% of all the solid tumours included in this report were so reviewed. The majority of the remainder were surgically inaccessible intracranial tumours. Subsequent biopsy and postmortem material, when available, is also collected and reviewed. Histology slides are retained by the Registry, allowing for revision of diagnoses with advances in knowledge. Detailed abstracts or photocopies are prepared from the hospital case notes and each case is followed up annually by writing to the clinician in charge, the general practitioner or occasionally direct to the parents. All the available clinical and pathological data are taken into account in making a final diagnosis. For leukaemias, clinical information is collected in the same way, and marrow reports by the respective hospital haematologists are accepted as proof of diagnosis. Postmortem material is obtained whenever possible. In most cases marrow biopsy specimens were seen by haematologists at the Royal Manchester Children's Hospital, which serves as the centre for paediatric oncology in the region covered by the Registry. In a few early cases the diagnosis of leukaemia was made on blood film and other clinical information alone. The tumours were classified by site and morphology according to the International Classification of Diseases for Oncology (ICD-O) (1976).

Average incidence rates per million person-years were calculated for each tumour type by dividing the numbers of cases by the sum of the estimates of the mid-year populations of children aged under 15 years resident in the NWRHA area (MRHB area before 1974). The total number of person-years was $24{\cdot}43 \times 10^6$. Trends in incidence were examined using a cusum technique (ICI Monograph, 1964). Median ages were estimated by ranking the cases in 6-month age groups and finding the age by which 50% of cases had occurred. The inter-quartile range represents the ages by which 25% and 75% of cases had occurred, and therefore includes 50% of all cases. For groups of less than 10 cases, age ranges or individual ages are shown in the tables instead of the inter-quartile range. Median ages were not calculated for mixed groups containing one or two cases of each of a number of histological entities, *e.g.* "other rare tumours". The binomial test was used to compare the sex ratio of children in each histological group with the ratio 1·1:1 in the study population.

### RESULTS

The total number of tumours included in this 24-year review was 2442, which gives an overall rate of 100 per million person-years (persons aged 0–14). The rates for individual tumour groups, therefore, also represent percentages of the total. Table I shows the distribution of tumours by primary site, grouped according to the main categories of the ICD-O topography section. By far the most common presenting sites for the solid tumours are those of the central nervous system. Lymph nodes, kidney, bone and soft-tissue tumours each comprise between 5% and 7% of the total. Tumours of lung, colon, bladder and breast are extremely rare in childhood.

TABLE I.—*Distribution of tumours by primary site*

| Site | No. | % Total |
|---|---|---|
| Haemopoietic and reticulo-endothelial systems | 873 | 35·7 |
| Brain and other central nervous system | 545 | 22·3 |
| Lymph nodes | 172 | 7·0 |
| Kidney | 147 | 6·0 |
| Connective and other soft tissue | 144 | 5·9 |
| Bone | 132 | 5·4 |
| Endocrine glands | 115 | 4·7 |
| Digestive organs and peritoneum | 97 | 4·0 |
| Eye | 94 | 3·9 |
| Genitourinary organs | 60 | 2·5 |
| Oral cavity and pharynx | 28 | 1·1 |
| Respiratory system and intrathoracic organs | 24 | 1·0 |
| Skin | 11 | 0·5 |
| | 2442 | 100·0 |

TABLE II.—*Leukaemia, lymphoma and other reticuloendothelial neoplasms*

| | Males | | Females | | | |
|---|---|---|---|---|---|---|
| Tumour type | No. | Median age in years (inter-quartile range) | No. | Median age in years (inter-quartile range) | Total | Rate per $10^6$ person-years |
| Acute lymphoid leukaemia + stem-cell leukaemia | 378 | 4½ (2½–8) | 260 | 4 (2½–7) | 638 | 26·1 |
| Acute myeloid leukaemia | 55 | 6½ (3–12½) | 66 | 7 (3½–10½) | 121 | 5·0 |
| Acute monocytic leukaemia | 6 | 11 (4½–13½)* | 14 | 2 (1–8) | 20 | 0·8 |
| Chronic myeloid leukaemia | 9 | 9 (1–14½)* | 4 | 10, 11½, 12, 12½† | 13 | 0·5 |
| Other leukaemia | 9 | — | 8 | — | 17 | 0·7 |
| Diffuse lymphocytic lymphoma | 64 | 6½ (4–8½) | 27 | 7½ (5½–10½) | 91 | 3·7 |
| Histiocytic lymphoma | 3 | 1½, 10, 11½† | 9 | 6½ (1–14)* | 12 | 0·5 |
| Other non-Hodgkin's lymphoma | 7 | — | 1 | — | 8 | 0·3 |
| Hodgkin's disease | 63 | 10½ (7–13) | 24 | 12½ (10–14) | 87 | 3·6 |
| Histiocytosis X | 35 | 1 (½–3) | 28 | 2 (½–6½) | 63 | 2·6 |
| Other reticuloendothelial neoplasms | 2 | — | 5 | — | 7 | 0·3 |

* Age range.

† Individual ages.

Tables II–V give a detailed breakdown of the main histological groups by sex, and specify median ages and rates.

*Table II: Leukaemia, lymphoma and other reticulo-endothelial neoplasms*

Acute leukaemias of lymphoid origin (ALL) represent over a quarter of all malignant neoplasms in this series. ALL presents most frequently in the age group 2–4 years. The diagnosis of "acute stem-cell leukaemia" is rarely made now, since with more specific staining techniques currently in use it is possible to classify these leukaemias. It is clear that the great majority of "stem-cell" leukaemias described in historical series are of lymphoid origin (Draper *et al.*, 1980; Hayhoe *et al.*, 1964; Shaw, 1976; Fernbach, 1977) and these are combined with acute lymphoid in the present report.

Acute myeloid leukaemia (AML), though rare by comparison with ALL, occurs with about the same frequency as such classical childhood tumours as neuroblastoma and Wilms' tumour. Acute monocytic leukaemia and chronic myeloid leukaemia are uncommon in childhood. There were no cases of chronic lymphoid leukaemia. Among the "other leukaemia" group were 6 cases of lymphosarcoma-cell leukaemia, 6 cases of erythroleukaemia, 2 of aleukaemic leukaemia and 3 of chloroma.

The most common sub-type of non-Hodgkin's lymphoma was diffuse lymphocytic lymphoma (82%) and the great majority of these were poorly differentiated. The other non-Hodgkin's lymphoma group includes 4 cases of nodular lymphocytic lymphoma, 2 cases described simply as non-Hodgkin's lymphoma which could not be classified further, 1 case of mycosis fungoides and 1 case of Burkitt's lymphoma. The latter occurred in a 2-year-old Caucasian boy, and Epstein–Barr virus antibodies were not detected. Non-Hodgkin's lymphoma tends to present in the 5–10-year age group. Hodgkin's disease is usually seen in older children, and the most common histological sub-groups were: mixed cellularity, 38 cases (44%) and lymphocyte-predominant, 28 cases (32%). Nodular sclerosing and lymphocyte-depleted were rarely seen, with 15 (17%) and 6 (7%) cases respectively.

It is now well established that the three syndromes—eosinophilic granuloma, Hand–Schüller–Christian disease and Letterer–Siwe disease—are closely related clinically and pathologically (Lichtenstein, 1953, 1964) and are collectively known as histiocytosis X. Though we believe that most cases of histiocytosis X are reported to the MCTR, ascertainment may not be as complete in this group as

TABLE III.—*Gliomas and other intracranial tumours*

| | Males | | Females | | | |
|---|---|---|---|---|---|---|
| Tumour type | No. | Median age in years (inter-quartile range) | No. | Median age in years (inter-quartile range) | Total | Rate per $10^6$ person-years |
| Juvenile astrocytoma | 63 | 6½ (4–10½) | 71 | 7 (4–11½) | 134 | 5·5 |
| Other astrocytoma | 47 | 7 (3–11) | 42 | 7½ (6–11) | 89 | 3·6 |
| Ependymoma | 30 | 3½ (2–7½) | 40 | 3½ (2–7½) | 70 | 2·9 |
| Medulloblastoma | 78 | 6 (3–9½) | 38 | 5½ (2–9½) | 116 | 4·8 |
| Other glioma | 13 | 5 (1–6½) | 9 | 6 (½–13)* | 22 | 0·9 |
| Craniopharyngioma | 13 | 9 (7–12) | 11 | 8 (5–10½) | 24 | 1·0 |
| Meningioma | 4 | < ½, < ½, 7, 14½† | 8 | 9 (½–14½)* | 12 | 0·5 |
| Other intracranial | 5 | — | 8 | — | 13 | 0·5 |
| Unbiopsied | 37 | — | 32 | — | 69 | 2·8 |

* Age range.

† Individual ages.

TABLE IV.—*Connective tissue tumours*

| | Males | | Females | | | |
|---|---|---|---|---|---|---|
| Tumour type | No. | Median age in years (inter-quartile range) | No. | Median age in years (inter-quartile range) | Total | Rate per $10^6$ person-years |
| Rhabdomyosarcoma | 58 | 4 (1½–7) | 37 | 3½ (2–6½) | 95 | 3·9 |
| Fibrosarcoma | 11 | 3 (1½–10) | 8 | 9½ (< ½–13½)* | 19 | 0·8 |
| Synovial sarcoma | 8 | 8½ (½–14)* | 1 | 11½† | 9 | 0·4 |
| Osteosarcoma | 27 | 12 (9½–14) | 37 | 12 (10–13½) | 64 | 2·6 |
| Ewing's | 25 | 9½ (6–11½) | 25 | 9½ (6½–12½) | 50 | 2·0 |
| Other connective tissue | 12 | — | 11 | — | 23 | 0·9 |

* Age range.

† Individual age.

among the frankly malignant neoplasms. Histiocytosis X frequently presents in children under 2 years old and may be congenital. The remaining 7 reticuloendothelial neoplasms were 5 cases of malignant histiocytosis and 2 microgliomas. Leukaemias, lymphomas and other reticuloendothelial neoplasms form nearly half of the total neoplasms in this study.

*Table III: Gliomas and other intracranial tumours*

Intracranial and other CNS tumours represent the largest group of solid tumours in childhood, and present at all ages throughout childhood. The most common type is the juvenile or pilocytic astrocytoma, which tends to be slow-growing and often of borderline malignancy. The "other astrocytoma" group consists mostly of tumours of varying cellularity with pleomorphic stellate and spindle astrocytes but no piloid bundles or microcysts. The group includes all grades between relatively hypocellular and highly cellular pleomorphic tumours. There were also 2 astroblastomas, 2 giant-cell astrocytomas and 2 gemistocytic astrocytomas. Meningioma infrequently presents in childhood and 8 of the cases in this series were considered benign. Cases of brain tumours which were clinically diagnosed were included only if there was positive evidence of an intracranial space-occupying lesion (*e.g.* by ventriculography or brain scan).

*Table IV: Connective-tissue tumours*

The most common type of connective-tissue tumour was rhabdomyosarcoma. There were 3 pleomorphic tumours, 4 which were of a highly differentiated myoblastic type and 14 alveolar. The remaining 74 cases were distributed equally

TABLE V.—*Embryonal and miscellaneous rare tumours*

| Tumour type | Males: No. | Males: Median age in years (inter-quartile range) | Females: No. | Females: Median age in years (inter-quartile range) | Total | Rate per $10^6$ person-years |
|---|---|---|---|---|---|---|
| Wilms' | 61 | 2½ (1–3½) | 63 | 2½ (1–4) | 124 | 5·1 |
| Other complex renal | 6 | — | 3 | — | 9 | 0·4 |
| Neuroblastoma | 91 | 2 (½–3½) | 67 | 2 (½–4) | 158 | 6·5 |
| Bilateral retinoblastoma | 14 | 1 (<½–2½) | 15 | ½ (<½–1½) | 29 | 1·2 |
| Unilateral retinoblastoma | 22 | 2 (1–3) | 22 | 2 (1–3) | 44 | 1·8 |
| Hepatoblastoma | 11 | 1 (½–4) | 2 | <½, ½† | 13 | 0·5 |
| Germinoma | 5 | 9 (<½–12½)* | 10 | 11½ (10½–12½) | 15 | 0·6 |
| Teratoma and yolk sac | 13 | 1½ (1–2) | 26 | 3½ (1½–12½) | 39 | 1·6 |
| Epithelial | 29 | 11½ (8½–13½) | 25 | 11½ (8½–13) | 54 | 2·2 |
| Unbiopsied extracranial | 8 | — | 11 | — | 19 | 0·8 |
| Other rare | 6 | — | 8 | — | 14 | 0·6 |
| Unclassified | 22 | — | 16 | — | 38 | 1·6 |

* Age range.

† Individual ages.

between loose embryonic (including botryoid) and dense embryonic types. Osteosarcoma and Ewing's tumour occurred with about equal frequency, and both present in later childhood, osteosarcoma typically at around puberty. The "other connective tissue tumour" group includes 6 cases of haemangiopericytoma, only 2 of which were frankly malignant, 3 leiomyosarcomas, 2 liposarcomas and 5 chondrosarcomas.

*Table V: Embryonal and miscellaneous rare tumours*

In addition to Wilms' tumour, 9 other complex renal tumours were seen—4 mesoblastic nephromas and 5 BMRTC (bone-metastasizing renal tumours of childhood, as described by Marsden & Lawler, 1978). Neuroblastoma is sometimes found by chance at necropsy, and 11 of the MCTR cases were identified in this way. Unilateral retinoblastoma was more common than bilateral and had a later median age of onset. Early onset in unilateral retinoblastoma may be an indication of its hereditary potential.

Germinoma, teratoma and yolk sac tumour can occur in combination, and pure malignant teratoma is very rare in childhood. The detailed histology of the MCTR series of germ cell tumours is discussed in detail elsewhere (Marsden & Birch, in preparation). Epithelial tumours, which are so frequent in adults, are extremely uncommon in childhood. The Manchester series includes 7 adrenal cortical carcinomas and 10 nasopharyngeal carcinomas. The "other rare tumours" group includes 3 cases of phaeochromocytoma which were histologically benign but in one case fatal.

*Sex distribution*

Overall there were significantly more males than females ($P < 0{\cdot}001$). Among individual groups, ALL, non-Hodgkin's lymphoma, Hodgkin's disease and medulloblastoma showed a marked excess of males, the difference being particularly great in Hodgkin's disease ($P$ in all cases $< 0{\cdot}001$). In hepatoblastoma the preponderance of males was significant at the 5% level. Germ cell tumours were the only neoplasms to show a significant excess of females ($P = 0{\cdot}02$ for teratomas and yolk sac tumours and $P = 0{\cdot}01$ for germinoma). The excess of males approached significance for rhabdomyosarcoma ($P = 0{\cdot}10$) and in osteosarcoma the female excess approached significance ($P = 0{\cdot}12$). There were no other marked differences.

*Incidence trends*

There was an overall increase in the annual incidence of childhood malignant disease during the study period. A large contributing factor to this upward trend was the significant increase in the incidence of ALL, which has already been reported (Birch *et al.*, 1979). There was no comparable change in the incidence of AML. Although there were too few cases of CML to establish a clear trend, 9 of the 13 cases occurred after 1970, and it may be that this disease is becoming more frequent. The only other group to show a significant increase with time was the epithelial. The germ cell tumours showed an upward trend which approached significance.

No other marked changes in incidence were observed. However, non-significant upward trends were seen in non-Hodgkin's lymphoma and Hodgkin's disease, and a non-significant downward trend among soft-tissue sarcomas. The incidence of all other tumour groups remained fairly constant throughout the study period.

### DISCUSSION

The MCTR is strictly population-based and ascertainment has been estimated to be 95–98% complete (Leck *et al.*, 1976). The extensive clinical and pathological information on each case ensures a high degree of diagnostic accuracy. The histology of many of the solid tumours was reviewed especially for the present study. Although the MCTR has actual histological material for only a minority of the leukaemias, marrow reports are available for the great majority of the remainder, and these, together with the clinical details, have been reviewed in the light of current knowledge. It is therefore believed that the present estimates reflect the true incidence of childhood malignancy in the North West region.

Accurate incidence data are important in the planning and evaluation of clinical trials. The number and relative proportions of various histological sub-types referred to a treatment centre may not reflect their distribution in the population. Survival rates for a group as a whole calculated from the results of such a trial may thus be distorted. In other studies also, *e.g.* epidemiological, social and psychological, where study of an entire population may not be possible, it is important to select a sample such that the various groups are neither over- nor under-represented. The present incidence data may be used for reference in the planning of such studies.

Most international data are presented using classifications based on site (*e.g.* Cancer Incidence in Five Continents, 1976). Whilst this is satisfactory for adult cancers where most are carcinomas, for malignant disease in children this produces a distorted picture. For example, cases of teratoma included in the MCTR presented at more than 20 different sites, rhabdomyosarcoma at over 30 sites, and neuroblastoma at more than 10 sites. These important groups are consequently "lost" when data are presented by site alone. International comparisons are therefore impossible and insights into aetiology may be missed. Nevertheless the pattern of primary sites seen in childhood is very different from that in adults, and the present study includes only one breast tumour, 4 malignant tumours of the colon and 6 malignant tumours of the lung. No tumours of the uterine cervix were seen.

Very few reliable population-based data which specify histology are available for comparison with the MCTR figures. The data from 2 recent studies based on the U.S. white population (Young & Miller, 1975) and the population of Sweden (Ericsson *et al.*, 1978) are remarkably similar to those of the present study. The distribution and rank order of the various tumour types in all 3 series are broadly the same. The differences which are seen, for example the lower incidence of rhabdomyosarcoma and Ewing's tumour and higher incidence of "other eye" tumours and histiocytic lymphoma in Sweden, may be the result of different interpretation of histology. Similarly the rather different distribution of CNS

tumours among the U.S. whites may be accounted for in the same way. In neither of these series was pathology the subject of a special review. The only 2 population-based series among non-Caucasians for which histological type is specified are described by Young & Miller (1975) for U.S. blacks and Hanawa (1975) for Japan. The main differences to note when comparing these data with those for Caucasians are the lower incidence of leukaemia and the absence of Ewing's tumour in U.S. blacks, and the high incidence of acute myeloid leukaemia and low incidence of CNS tumours among Japanese children. It would be interesting to compare the incidence among black African populations with the U.S. blacks and American Japanese migrants with the native Japanese, in order to evaluate the effects of migration on incidence among different ethnic groups. As yet these data are not available.

Perhaps the most striking features to emerge in comparing the available series are their similarities rather than their differences; unlike adult tumours where very wide variations are seen between different parts of the world (Doll, 1977). However, it is apparent that some ethnic differences do exist in children's tumours and there is an obvious need for good-quality data from other parts of the world in order to establish a clear pattern. For more detailed discussion of the variations in incidence of childhood malignancy among different populations see Birch (1979), Draper *et al.* (1980) and Davies (1976).

Few studies on trends in incidence of childhood malignancy have been carried out, since few registries have been established long enough to make such studies worthwhile. Reports from both Sweden (Ericsson *et al.*, 1978) and Finland (Teppo *et al.*, 1975) have demonstrated significant increases in the incidence of CNS tumours, and in Sweden the incidence of neuroblastoma and Wilms' tumour also rose during the period 1958–74. In neither of these studies was ascertainment estimated nor was the histology specially reviewed. It is possible that the reported trends in incidence may in part at least reflect changes in ascertainment and diagnostic fashion. However, if it is assumed that these figures represent genuine incidence trends, then interesting comparisons may be drawn between these data and those of the present study.

An increase in the incidence of ALL in north-west England, which apparently began around 1970, has been shown (Birch *et al.*, 1979). No overall increase was found in Sweden and Finland, although the Swedish data show increases in girls aged 0–4 years and boys aged 5–9 years, with decreases in older children. In the MTCR series the increase is concentrated in the 1–5-year age group. The Finnish data were not analysed by separate age groups. Neither in the Swedish nor the Finnish studies were different cell types considered separately. The average annual incidence of leukaemia in these countries is higher than that estimated by the MCTR. It may be, therefore, that the incidence in north-west England is rising to a level already established in Sweden and Finland, and any environmental factors responsible for this increase may have been active there for some time. The rise in the incidence of ALL in north-west England is currently being studied in detail and we hope to publish our results in the near future. No change in the incidence of CNS tumours, neuroblastomas and Wilms' tumour was seen in the MCTR series. The incidence of these tumours in Sweden is higher than in north-west England and it may be that whatever factors caused the increase in Sweden are not present in this region.

The rise in the incidence of epithelial tumours in the present series suggests that environmental carcinogens of importance in the induction of carcinomas in adults following chronic exposure may have increased. This could lead to earlier onset of tumours as a result of more rapidly accumulated doses. Epithelial tumours included in the current study presented mainly in children in the 10–14-year age group. It would be pertinent to examine trends in the incidence of epithelial tumours in young adults in the North West region and to compare the incidence of these tumours in both age groups with that in other regions.

The increase in the incidence of germ cell tumours has been the subject of a separate study, and is discussed elsewhere (Birch *et al.*, in preparation). However, the detection of this increase illustrates the importance of histological review by experts. These tumours are difficult to classify and have been subject to changes in classification and nomenclature in recent years. Because the material was specially reviewed for the present study our results are not influenced by these changes. These tumours occur at many different sites, and trends in their incidence cannot therefore be studied using classifications based on site alone.

A feature of many childhood tumours is male preponderance. Some of the male excess seen in certain adult tumours may be accounted for by smoking habits and exposure to carcinogens at work. Such an explanation cannot account for the sex distribution seen among cases of childhood cancer. The male excess is particularly marked in neoplasms of lymphoid origin, which represent over a third of all tumours. Perhaps maternal immunological mechanisms result in susceptibility of the offspring to the induction of lymphoid neoplasia, and the male foetus is at a particular disadvantage. The female excess seen in germ cell tumours can, in part, be accounted for by the earlier onset of ovarian tumours in girls than testicular tumours in boys. Many boys develop their tumours after the age of 15 and therefore are excluded from the Registry. However, sacrococcygeal tumours occur almost exclusively in girls. No explanation for this is immediately apparent, though it has been suggested that this sex difference may be due to the longer period of differentiation which genital cells arising from the primitive knot (located in the region of the coccyx) undergo in formation of the ovary compared with the testis. Thus the genital cells may be at a greater risk of becoming ectopic and giving rise to a teratoma in the female than in the male (Gross *et al.*, 1951). Osteosarcoma tends to occur at around the time of puberty, and earlier onset of puberty in girls explains the female excess seen in this tumour in the under-15 group.

Malignant disease is exceeded only by accidents as a cause of death in the 1–14-year age group. It is nevertheless rare, occurring in about 1 in 10,000 children each year. A wide range of histological types can occur at virtually any site in the body. It is, therefore, inappropriate to use classifications based on site, and the rarity and complexity of some of these tumours demonstrates the necessity of special pathological review. The study of incidence trends among childhood tumours requires a very long time series with consistent histological classification. The MCTR data are unique in their accuracy, completeness and extent, and are therefore highly suitable for such studies. Although the present analyses show marked increases in the incidence of 3 tumour types, most groups showed no change over the 24-year study period. Changes in incidence should alert workers to possible changes in environmental influences and form the basis for further study. Uniform incidence of tumours over a long time might indicate that environmental factors were of little aetiological significance and that genetic influences predominated. If factors in the environment are of importance in the induction of these tumours, these factors must be evenly distributed with time.

The early onset (for most childhood tumours onset is usually under the age of 5 years), and the embryonal nature of the major paediatric tumour groups, suggest a pre-natal origin, and genetic factors, immunological influences *in utero*, pregnancy infections and exposure to drugs and other chemicals *in utero* may be important. In order to explore such factors of potential aetiological significance and to investigate further the increases in incidence of some tumours, a prospective case/control study has been initiated. The mothers' experiences during pregnancy, associations between tumours and congenital abnormalities, diseases in other family members and exposure of the child to potential carcinogens will be studied in detail. By the monitoring of incidence, analysis of histology and age characteristics, accurate tumour registry data not only give rise to aetiological clues which can be explored, but also supply essential information for the setting up of clinical trials and planning of services for children with cancer. More data based on histology are needed from different parts of the world, so that international comparisons can be made.

We are grateful for the advice of Dr O. G. Dodge and all previous members of the panel of pathologists associated with the University of Manchester Children's Tumour Registry.

We would also like to thank the North Western Regional consultants, general practitioners and medical records officers who have so willingly contributed information and material to the Registry.

The work described in this report was supported by a three-year grant from the US Public Health Services (Research Grant No. CA 14992) and grants from Cancer Research Campaign and the North Western Regional Health Authority.

### REFERENCES

BIRCH, J. M. (1979) The epidemiology of childhood tumours. In *Topics in Paediatrics I, Haematology and Oncology*. Ed. Morris Jones. Tunbridge Wells: Pitman Medical Publishing. p. 1.

BIRCH, J. M., MARSDEN, H. B. & SWINDELL, R. (1979) Incidence of acute leukaemia of childhood in North West England. *Lancet*, ii, 854.

DAVIES, J. N. P. (1976) Some variations in childhood cancers throughout the world. *Recent Results in Cancer Res.*, **13**, 28.

DOLL, R. (1977) Strategy for detection of cancer hazards to man. *Nature*, **265**, 589.

DRAPER, G. J., BIRCH, J. M., BITHELL, J. F. & 6 others (1980) *Childhood Cancer in Britain* 1953–1975. London: HMSO. (In press.)

ERICSSON, J. L.-E., KARNSTRÖM, L. & MATTSSON, B. (1978) Childhood Cancer in Sweden, 1958–1974. I. Incidence and mortality. *Acta Paediatr. Scand.*, **67**, 425.

FERNBACH, D. J. (1977) Natural history of acute leukaemia. In *Clinical Pediatric Oncology*. Eds Sutow *et al.* St Louis: C. V. Mosby Co. p. 153.

GROSS, R. E., CLATWORTHY, H. W. & MECKER, I. A. (1951) Sacrococcygeal teratomas in infants and children: A report of 40 cases. *Surg. Gynecol. Obstet.*, **92**, 341.

HANAWA, Y. (1975) *All Japan Children's Cancer Registration, 1969–73.* Tokyo: Children's Cancer Association of Japan.

HAYHOE, F. G. J., QUAGLINO, D. & DOLL, R. (1964) The cytology and cytochemistry of acute leukaemias: A study of 140 cases. *MRC Special Report Series No.* 304. London: HMSO.

*International Classification of Diseases for Oncology* 1st Ed. (1976). Geneva: WHO.

ICI Monograph No. 3 (1964) *Cumulative Sum Techniques.* Edinburgh: Oliver & Boyd.

LECK, I., BIRCH, J. M., MARSDEN, H. B. & STEWARD, J. K. (1976) Methods of classifying and ascertaining children's tumours. *Br. J. Cancer*, **34**, 69.

LICHTENSTEIN, L. (1953) Histiocytosis X. Integration of eosinophilic granuloma of bone, "Letterer–Siwe disease" and "Schüller–Christian disease" as related manifestations of a single nosologic entity. *Arch. Pathol.*, **56**, 84.

LICHTENSTEIN, L. (1964) Histiocytosis X (eosinophilic granuloma of bone, Letterer–Siwe disease and Schüller–Christian disease) *J. Bone Joint Surg.*, **46-A**, 76.

MARSDEN, H. B. & LAWLER, W. (1978) Bone-metastasizing renal tumour of childhood. *Br. J. Cancer*, **38**, 437.

MARSDEN, H. B. & STEWARD, J. K. (1976) (Eds). Tumours in Children. *Recent Results in Cancer Res.*, **13**.

SHAW, M. T. (1976) The cytochemistry of acute leukaemia: A diagnostic and prognostic evaluation. *Semin. Oncol.*, **3**, 219.

TEPPO, L., SALONEN, T. & HAKULINEN, T. (1975) Incidence of childhood cancer in Finland. *J. Natl Cancer Inst.*, **55**, 1065.

WORLD HEALTH ORGANIZATION (1976) Cancer Incidence in Five Continents Vol. III. Lyon: IARC Scientific Publications.

YOUNG, J. L. & MILLER, R. W. (1975) Incidence of malignant tumours in US children. *J. Pediatr.*, **86**, 254.